# Temporal Trends of Influenza A (H1N1) Virus Seroprevalence following 2009 Pandemic Wave in Guangdong, China: Three Cross-Sectional Serology Surveys

**DOI:** 10.1371/journal.pone.0038768

**Published:** 2012-06-18

**Authors:** Fen Yang, Jianfeng He, Haojie Zhong, Changwen Ke, Xin Zhang, Teng Hong, Hanzhong Ni, Jinyan Lin

**Affiliations:** Guangdong Provincial Center for Disease Control and Prevention, Guangzhou City, Guangdong Province, People’s Republic of China; National University of Singapore, Singapore

## Abstract

**Background:**

To evaluate the temporal trends of seroprevalence to pH1N1 among the Guangdong population following 2009 H1N1 pandemic wave, we conducted three cross-sectional serology surveys in 2010.

**Methodology/Principal Findings:**

Three surveys were carried out consecutively in 2010 from January 8 to January 24, from March 15 to April 10 and from August 23 to September 4. Sample populations comprising of 4725, 4727, and 4721 subjects respectively were randomly selected for study in these three surveys. The level of antibodies against pH1N1 was evaluated by hemagglutination inhibition assay. In survey 1, the seroprevalence of pH1N1 among all the subjects is 25.1%, declining to 18.4% in survey 2 and increasing to 21.4% in survey 3. Among vaccinated subjects, the seroprevalence was 49.0%, 53.0%, and 49.4% in the three consecutive surveys, showing no significant differences. In contrast, among non-vaccinated subjects, the seroprevalence declined significantly from 22.8% (survey 1) to 14.3% (survey 2) and subsequently increased to 18.1% (survey 3). The multivariate logistic regression analysis revealed that seroprevalence to pH1N1 in non-vaccinated individuals correlated with the investigated order of the surveys, age, and region (all P<0.05). However, it was not correlated with gender (P = 0.650), seasonal influenza vaccination history (P = 0.402) and symptoms (P = 0.074).

**Conclusions/Significance:**

In Guangdong, the seroprevalance to pH1N1 decreased initially and then rebounded modestly during the first 9 months following the 2009 pandemic wave. Our results suggest that the prevalence of pH1N1 is still correlated with age and population density during the post-pandemic period. An early end to the free pH1N1 vaccination program might be another important reason for the slight rebound in seroprevalance. Our study findings can help the Guangdong authorities to make evidence-based decisions about a long-term vaccination strategy and boost immunity in specific population groups (such as children and people living in the capital-city) to prevent further transmission in the future.

## Introduction

In April 2009, a novel influenza virus strain of subtype A (H1N1) first emerged in the United States [1] and Mexico [2], later causing a worldwide pandemic. On June 11, 2009, World Health Organization (WHO) declared a global pandemic for the first time in the last 41 years [3], [4]. In China, the first case of pandemic H1N1 (pH1N1) was detected on April 30, 2009. In Guangdong province (a province located in southern China, a semi-tropical region in Southeast Asian with a population of 100 million), the first case of pH1N1 was reported on May 18, 2009. From May 1, 2009 to December 31, 2009, a total of 9896 laboratory confirmed cases and 36 pH1N1 deaths were reported to the Guangdong Notifiable Disease Database. Virological surveillance documented sustained and widespread community transmission since early October 2009, followed by a single epidemic wave which peaked in late November 2009 and subsided by the end of December 2009 [5].

Despite intensive surveillance for infected cases during the pandemic [6], it is still very likely that the case reports underestimated the true infection rate in the population [7], [8] due to the mis-counting of the asymptomatic and mild cases. Many studies have been performed to examine the seroprevalence in order to obtain more accurate evaluations of the true infection rates [9], [10], although few of them were aimed at tracking the temporal trends of the seroprevalence in the pandemic. As all pandemics in history are different and the temporal trends in one may not be the case in other pandemics [11], research on the temporal fluctuations of pH1N1 is important to give a comprehensive insight into the transmission feature throughout the duration of the pandemic. The purpose of this study is to understand the impact of the 2009 winter wave of the pH1N1 epidemic and the effect of the free pH1N1 vaccination program implemented from October 2009 in Guangdong, to evaluate the risk of recurrence in the 2010 summer wave, and to explore underlying influencing factors. We conducted three consecutive serological surveys on randomly selected sample populations from Guangdong in January, March-April and August-September of 2010 respectively. Combining the findings from these three surveys will provide valuable information about the likelihood of potential recurrence and future outbreaks. It will guide us in formulating vaccination and treatment strategies during the post-pandemic period. WHO Director-General Dr. Margaret Chan announced on August 10, 2010 that the global H1N1 influenza virus had moved into the post-pandemic phase [12], [13].

## Methods

### Investigation Date

**Figure 1:**
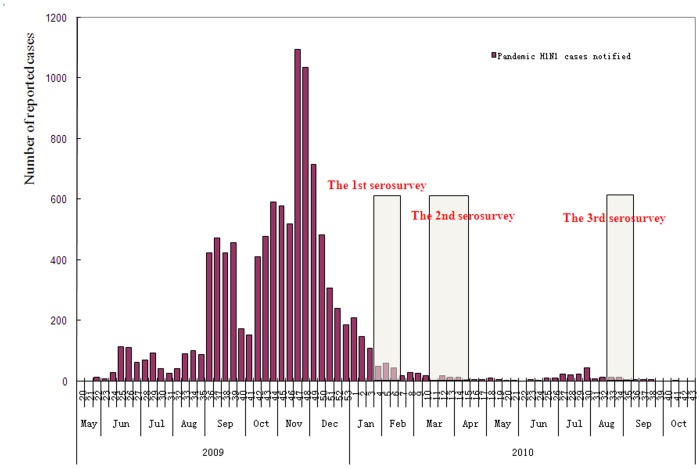
Sampling period for three serosurveys shown relative to epidemic curve in Guangdong China. Three surveys were consecutively carried out in 2010 during 9 months following the 2009 pandemic wave in Guangdong, China. Survey 1 were conducted from January 8 to January 24, survey 2 from March 15 to April 10 and survey 3 from August 23 to September 4.

The first survey was conducted from January 11 to January 24, 2010. As the 2009 winter epidemic wave of pH1N1 continued from early October to late December, serology investigation in January 2010 could help to estimate the extent of infection during this wave. Considering that the summer epidemic wave of influenza in Guangdong province usually happens between March and August [14], the second and third surveys were performed in March- April (from March 15 to April 10) and August-September (from August 23 to September 4) 2010 to capture the possible second wave of the pandemic. (Figure 1) Combining the findings of these three surveys, we would be able to ascertain a better understanding of the epidemic characteristics of pH1N1.

### Sample Age Groups

The target population fell into five age groups representative of five categories of people in Guangdong province: toddlers and preschoolers (0∼5 years old), school-age children (6∼15 years old), teens and young adults (16∼25 years old), working-age adults (26∼60 years old ) and retired or older adults (60∼years old). Each group has a specific lifestyle and is likely to experience a different risk of pH1N1 infection.

### Sampling Procedures

**Figure 2:**
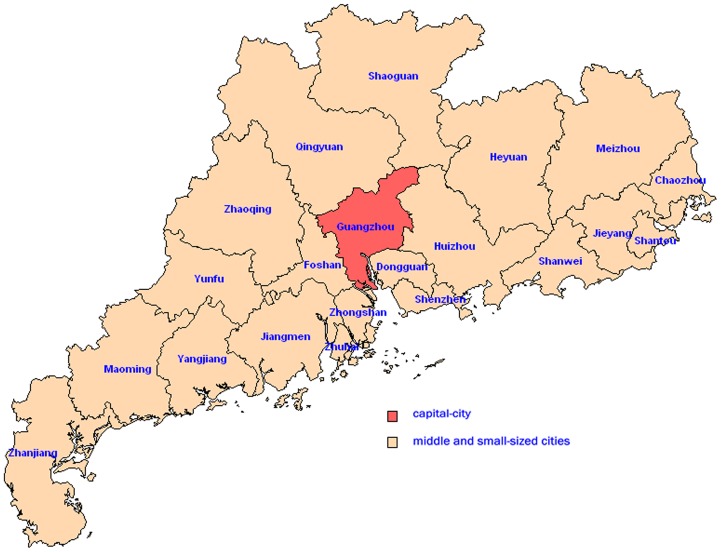
Twenty-one cities of Guangdong province were applied by a multi-stage stratified and cluster random sampling for sample selection in each survey. Guangdong province, including one capital-city (Guangzhou city) and twenty middle and small-sized cities, was divided into three population strata: a) the core area of the capital city, b) prefectures of other urban areas and c) prefectures of rural areas. At least two districts in each of the three population strata were randomly selected.

Based on the residency address, a multi-stage stratified and cluster random sampling was applied for sample selection in each survey [15]. Guangdong province, including one capital-city (Guangzhou city) and twenty middle and small-sized cities (Figure 2), was divided into three population strata: a) the core area of the capital city, b) prefectures of other urban areas and c) prefectures of rural areas. We were instructed to randomly select at least two districts in each of the three population strata [16]. By using a random digits table, five urban districts from the capital-city and twenty districts/counties from twenty middle and small-sized cities (one district/county per middle and small-sized city) were randomly selected and remained unchanged in the three surveys. Then at least one street/town in each district/county and finally at least one community/village in each street/town were chosen. Once the communities/villages were selected, the investigating team obtained a name list of all individuals (including age) residing in the communities/villages and randomly selected individuals from each sampling age group. A sample size of 300 from each age group in each population strata was the target to enroll for each survey. The selected study subjects were asked by the investigators whether they would like to participate in the study. If a selected individual declined to participate, the next individual on the list was approached and asked to participate. Informed consent had to be obtained from each study participant before an interview. Informed consent was provided by adults (≥18 years) themselves. Assent was provided by adolescents (10∼17 years), themselves, and informed consent was given by a parent or a legal guardian of the adolescent. Informed consent for children (<10 years) was provided by a parent or a legal guardian. The survey questionnaire was completed by a trained interviewer. Blood samples were collected during a face-to-face interview. The questionnaire included information on the subject’s age, gender, occupation, vaccination history of seasonal inﬂuenza (since 2007) and pH1N1, presence/absence of flu-like illnesses (since May 2009). In total, 4745 study subjects participated in the first survey, 4773 study subjects participated in the second survey and 4732 study subjects participated in the third survey. Excluding the subjects with incomplete questionnaires and invalid blood samples, we finally obtained 4725, 4727 and 4721 valid subjects in the three surveys.

### Sample Size

The seroprevalence of pH1N1 was approximately 25% in China according to sentinel surveillance. With a 95% confidence interval of +/− 10% (15–35%), we calculated the sample size of 300 from each age group in each of the three strata for an expected sample size, totaling 4500 subjects for each survey.

### Laboratory Methods

Antibodies against the pH1N1 virus were detected by haemmagglutination inhibition (HI) assay using 0.5% turkey red blood cells, according to the standard methods provided at WHO National Influenza Centres [8], [17].The human serum samples were first treated with receptor-destroying enzyme (RDE) provided by Chinese National Influenza Center (CNIC) with a ratio of 4∶1 (vol/vol) at 37°C overnight to eliminate nonspecific inhibitors of hemagglutination. The samples were then incubated at 56°C for 30 min to inactivate RDE followed by testing for HA-specific antibodies using the standard hemagglutination inhibition (HI) assay with A/California/7/2009 employed as antigens. Ten different dilutions (1∶5, 1∶10, 1∶20, 1∶40, 1∶80, 1∶160, 1∶320, 1∶640, 1∶1280, 1∶2560) of serum samples were analyzed in virus-specific assays to evaluate the HA-specific antibody titer. Serum-only control for each human serum sample without addition of viral antigen was also assayed in parallel with the virus-specific assays. Only the virus-specific assays with titer values greater than or equal to the corresponding serum-only control values were considered valid. Samples with HI titers ≥1∶40 were considered as seropositive.

### Ethics Statement

This study was approved by the ethics committee of the Guangdong Center for Disease Control and Prevention and written informed consent was obtained from the study subjects.

### Statistical Analysis

A standard database was created in EPI Data software (version 3.02). The survey questionnaires were inputted twice into the database and checked for consistency. Statistical analyses were performed using SPSS 13.0 software (SPSS Inc., Chicago, IL, USA). The seroprevalence was defined as the percentage of serum titers ≥40 by HI. Frequencies and proportions were calculated to describe the demographic distributions for the three serosurveys. The chi-square test was used to compare the differences in the seroprevalence among different groups between each two serosurveys. The significance level was set as 0.018 to give an overall p-value of 0.05. To control for possible confounders, multivariable logistic regression analyses was performed. The dependent variable was the presence of pH1N1 seropositivity vs. no seropositivity. The examined independent variables were the investigation order of the surveys, gender, age, occupation, region (capital city or rural vs. other urban areas), seasonal influenza vaccination history and symptoms. The odds ratio (OR) and 95% confidence interval (95% CI) were calculated for each risk factor. Significance (P<0.05) was based on Likelihood Ratio test. The final model examining risk factors for pH1N1 infection included the investigation order of the surveys, age, and region. The occupation group was excluded from the model because of the collinear relationship with age (p<0.001).

## Results

### Characteristics of Study Subjects

The demographic characteristics of the sampled subjects changed slightly in the three consecutive surveys, as presented in Table 1. The median age was 28.3 (range, 1–84) for survey 1, 28.6 (range, 1–86) for survey 2, and 28.2 (range, 1–84) for survey 3. Male to female ratio was 1∶1 in all three surveys. In the three consecutive surveys respectively, about 8.6% (408/4725), 10.7% (508/4727), and 10.7% (506/4721) of subjects reported receiving the pH1N1 vaccine since October 2009.

**Table 1:** Demographic characteristics of the subjects in the three surveys.

	Survey 1		Survey 2		Survey 3	
	n	%	n	%	n	%
**Age groups (years)**						
0∼5	777	16.4	658	13.9	913	19.3
6∼15	1100	23.3	1203	25.4	986	20.9
16∼25	991	21.0	972	20.6	963	20.4
26∼60	963	20.4	976	20.6	1054	22.3
>60	894	18.9	918	19.4	805	17.1
**Gender**						
Male	2338	49.5	2215	46.9	2318	49.1
Female	2387	50.5	2512	53.1	2403	50.9
**Urban/rural**						
Capital-city	1525	32.3	1527	32.3	1524	32.3
Other urban areas	1679	35.5	1600	33.8	1521	32.2
Rural areas	1521	32.2	1600	33.8	1676	35.5
**Occupation**						
Children scattered	252	5.3	231	4.9	294	6.2
Children in kindergartens	780	16.5	768	16.2	728	15.4
Student	1298	27.5	1264	26.7	1278	27.1
Teacher	161	3.4	174	3.7	96	2.0
Medical personnel	286	6.1	247	5.2	224	4.7
Other	1948	41.2	2043	43.2	2101	44.5
**Inoculated with pH1N1 vaccine**						
Yes	408	8.6	508	10.7	506	10.7
No	4317	91.4	4219	89.3	4215	89.3
***Total Number of Cases***	**4725**	**100.0**	**4727**	**100.0**	**4721**	**100.0**

### Temporal Trends of the Seroprevalence in Guangdong

Seroprevalence of pH1N1 in Guangdong declined significantly from 25.1% (95% CI: 23.9–26.3) to 18.4% (95% CI: 17.3–19.5) between survey 1 and 2, then increased significantly to 21.4% (95% CI: 20.2–22.6) in survey 3. (Table 2).

**Table 2:** Pandemic H1N1 seroprevalence in the vaccinated and non-vaccinated people, Guangdong, 2010.

	No. Tested (seroprevalence%, 95% CI)		
	Survey 1	Survey 2	Survey 3
total	4725(25.1, 23.9–26.3)	4727(18.4,17.3–19.5)*	4721(21.4,20.2–22.6)# *
Vaccinated	408(49.0,44.1–53.9)	508(53.0,48.7–57.3)	506(49.4,45.0–53.8)
Non-vaccinated	4317(22.8,21.5–24.1)	4219(14.3,13.2–15.4)*	4215(18.1,16.9–19.3)# *

CI:Confidence Interval;

* compared with survey1,P<0.05(by Chi-square);

# compared with survey2, P<0.05(by Chi-square);

### Temporal Trends of the Seroprevalence among Vaccinated Subjects

For subjects who reported receiving the pH1N1 vaccine (vaccinated subjects), the seroprevalence was 49.0% (95% CI: 44.1–53.9), 53.0% (95% CI: 48.7–57.3), 49.4% (95% CI: 45.0–53.8) in the three consecutive surveys, respectively. No significant differences across serosurveys were observed. By comparing the temporal trends of the seroprevalence with other different positive titers (HI titer ≥ 1∶20, 1∶80, 1∶160), we found that the seroprevalence with titer ≥ 1∶80 and 1∶160 dropped noticeable between survey 2 and 3 (Not shown).

When stratifying by gender, age and region (capital city or rural vs. other urban areas), the stability of the seroprevalence (HI titer ≥ 1∶40) across the three surveys persisted, except for two different strata of region (capital-city and other urban groups). Among the capital-city group, the seroprevalence increased significantly from 38.6% (95% CI: 35.1–42.1) to 51.1% (95% CI: 48.0–54.2) between survey 1 and 2, but showed no change between survey 2 and 3. The seroprevalence among the urban group showed no difference between survey 1 and 2, but declined significantly from 53.0% (95% CI: 48.9–57.1) to 39.1% (95% CI: 35.2–43.0) between survey 2 and 3 (Table 3). By comparing the temporal trends of the seroprevalence with other different positive titers (HI titer ≥ 1∶20, 1∶80, 1∶160), we found that the seroprevalence with HI titer ≥ 1∶160 in the capital-city group have been a noticeable drop between survey 2 and 3 (Not shown).

**Table 3:** Temporal trends of 2009 H1N1 seroprevalence by demographic characterstics among the vaccinated subjects,Guangdong, 2010.

	No. Tested (seroprevalence%, 95% CI)		
	Survey 1	Survey 2	Survey 3
**Gender**			
Male	181(51.3, 44.0–58.6)	218(50.9, 44.3–57.5)	245(46.5, 40.3–52.7)
Female	227(47.1, 40.6–53.6)	290(54.4, 48.7–60.1)	261(52.1, 46.0–58.2)
**Age group (years)**			
0∼5	25(32.0, 13.7–50.3)	14(42.8, 16.9–68.7)	48(43.7, 29.7–57.7)
6∼15	108(52.7, 43.3–62.1)	143(50.3, 42.1–58.5)	167(45.5, 37.9–53.1)
16∼25	117(49.5, 40.4–58.6)	149(63.7, 56.0–71.4)	151(57.6, 49.7–65.5)
26∼60	136(50.7, 42.3–59.1)	155(49.0, 41.1–56.9)	110(48.1, 38.8–57.4)
>60	22(36.3, 16.2–56.4)	47(42.5, 28.4–56.6)	30(43.3, 25.6–61.0)
**Urban/rural**			
Capital-city	194(38.6, 31.7–45.5)	256(51.1, 45.0–57.2)*	249(53.8, 47.6–60.0) *
Other urban areas	137(59.8, 51.6–68.0)	149(53.0, 45.0–61.0)	156(39.1, 31.4–46.8)# *
Rural areas	77(55.8, 44.7–66.9)	103(57.2, 47.6–66.8)	101(54.4, 44.7–64.1)

CI:Confidence Interval;

* compared with survey1,P<0.05(by Chi-square);

# compared with survey2, P<0.05(by Chi-square);

### Temporal Trends of the Seroprevalence among Non-vaccinated Subjects

For subjects who reported not receiving the pH1N1 vaccine (non-vaccinated subjects), the seroprevalence declined significantly from 22.8% (95% CI: 22.2–23.4) to 14.3% (95% CI: 13.8–14.8) between survey 1 and 2, then increased significantly to 18.1% (95% CI: 17.5–18.7) in survey 3. Seroprevalence with other different positive titers (HI titer ≥ 1∶20, 1∶80, 1∶160) showed similar temporal trends.

When stratifying by gender, age, occupation, region, seasonal influenza vaccination history and symptoms, the decline of the seroprevalence persisted between survey 1 and 2, except for three different strata of occupation (children scattered, teachers and medical personnel). No significant differences were observed among these three strata of occupation (all P>0.018). Between survey 2 and 3, the increase of the seroprevalence persisted among different gender groups, three age groups (0∼5, 26∼60, >60 years), the capital-city group, children in kindergartens, subjects with other occupations, the seasonal vaccination group and people without flu-like symptoms (all P<0.018) (Table 4).

**Table 4:** Temporal trends of 2009 H1N1 seroprevalence by inﬂuencing factors among the non-vaccinated subjects, Guangdong, 2010.

	No. Tested (seroprevalence%, 95% CI)		
	Survey 1	Survey 2	Survey 3
**Gender**			
Male	2157(22.1, 20.3–23.9)	1997(15.1, 13.5–16.7)*	2073(18.3, 16.6–20.0)#
Female	2160(23.4, 21.6–25.2)	2222(13.5, 12.1–14.9)*	2142(17.7, 16.1–19.3)#
**Age group (years)**			
0∼5	752(28.1, 24.9–31.3)	644(14.4, 11.7–17.1)*	865(19.6, 17.0–22.2)#
6∼15	992(28.6, 25.8–31.4)	1060(19.6, 17.2–22.0)*	819(23.5, 20.6–26.4)#
16∼25	874(28.6, 25.6–31.6)	823(21.8, 19.0–24.6)*	812(20.9, 18.1–23.7)
26∼60	827(15.9, 13.4–18.4)	821(7.7, 5.9–9.5)*	944(14.4, 12.2–16.6)#
>60	872(12.2, 10.0–14.4)	871(6.7, 5.0–8.4*	775(11.8, 9.5–14.1)#
**Urban/rural**			
Capital-city	1331(27.3, 24.9–29.7)	1271(14.1, 12.2–16.0)*	1275(25.4, 23.0–27.8)#
Other urban areas	1542(21.4, 19.4–23.4)	1451(14.9, 13.1–16.7)*	1365(16.7, 14.7–18.7)
Rural areas	1444(20.1, 18.0–22.2)	1497(13.7, 12.0–15.4)*	1575(13.2, 11.5–14.9)
**Occupation**			
Children scattered	245(24.0, 18.7–29.3)	230(15.6, 10.9–20.3)	286(18.8, 14.3–23.3)
Children in kindergartens	757(26.1, 23.0–29.2)	738(13.5, 11.0–16.0)*	686(19.3, 16.3–22.3)#
Student	1169(33.0, 30.3–35.7)	1077(24.5, 21.9–27.1)*	1037(25.3, 22.7–27.9)
Teacher	139(22.3, 15.4–29.2)	140(13.5, 7.8–19.2)	87(20.6, 12.1–29.1)
Medical personnel	105(33.3, 24.3–42.3)	101(22.7, 14.5–30.9)	119(20.1, 12.9–27.3)
Other	1902(14.5, 12.9–16.1)	1933(8.3, 7.1–9.5)*	2000(13.4, 11.9–14.9)#
**Vaccination history for seasonal influenza**			
Yes	461(25.8, 21.8–29.8)	369(14.6, 11.0–18.2)*	309(22.9, 18.2–27.6)#
No	3856(22.4, 21.1–23.7)	3850(14.2, 13.1–15.3)*	3906(17.6, 16.4–18.8)#
**With flu-like symptoms**			
Yes	2491(23.9, 22.2–25.6)	2311(14.4, 13.0–15.8)*	2010(16.9, 15.3–18.5)#
No	1826(21.3, 19.4–23.2)	1908(14.2, 12.6–15.8)*	2205(19.0, 17.4–20.6)#

CI:Confidence Interval;

* compared with survey1,P<0.05(by Chi-square);

# compared with survey2, P<0.05(by Chi-square);

The multivariate logistic regression analysis revealed that the seroprevalence for pH1N1 correlated with the investigated order of the surveys, age and region (all P<0.05). However, it was not correlated with gender (P = 0.650), seasonal influenza vaccination history (P = 0.402) and symptoms (P = 0.074). The occupation group was excluded from the final regression model because they had a high correlation with age (P<0.001).

The odds of pH1N1 seropositivity in survey 2 (OR: 0.55, 95% CI: 0.50–0.62) and survey 3 (OR: 0.75, 95% CI: 0.67–0.83) were significantly lower than the odds of seropositivity in survey 1. The odds in survey 3 was significantly higher when compared with that of survey 2. The odds of pH1N1 seropositivity in the 6∼15(OR: 1.20, 95% CI: 1.04–1.37) and 16∼25 (OR: 1.17, 95% CI: 1.02–1.35) age groups were significantly higher when compared with the odds of seropositivity in the 6∼15 age group. However, the odds of pH1N1 seropositivity in the 26∼60 (OR: 1.20, 95% CI: 1.04–1.37) and >60 (OR: 1.17, 95% CI: 1.02–1.35) age groups were significantly lower when compared with the odds of seropositivity in the 6∼15 age group. The odds of pH1N1 seropositivity in the urban areas (OR: 0.73, 95% CI: 0.66–0.82) and the rural areas (OR: 0.79, 95% CI: 0.69–0.90) were significantly lower when compared with the odds of seropositivity in the capital city. There was no statistically significant difference in the odds of seropositivity between urban and rural areas (Table 5).

**Table 5:** Logistic regression analysis of factors associated with 2009 H1N1 seroprevalence among the non-vaccinated subjects, Guangdong, 2010.

	B	OR(95%CI)	P
**Investigation order of the surveys**			
Survey 1		1	
Survey 2	−0.59	0.55(0.50–0.62)	<0.001
Survey 3	−0.29	0.75(0.67–0.83)	<0.001
**Gender**			
Male	−0.02	0.98(0.89–1.07)	0.650
Female		1	
**Age group (years)**			
0∼5		1	
6∼15	0.18	1.20(1.04–1.37)	0.010
16∼25	0.16	1.17(1.02–1.35)	0.027
26∼60	−0.61	0.54(0.46–0.64)	<0.001
>60	−0.88	0.41(0.35–0.49)	<0.001
**Region**			
Capital-city		1	
Other urban areas	0.31	0.73(0.66–0.82)	<0.001
Rural areas	0.46	0.63(0.56–0.71)	<0.001
**Vaccination history for seasonal influenza**			
Yes	−0.07	0.93(0.80–1.10)	0.402
No		1	
**With flu-like symptoms**			
Yes	−0.08	0.92(0.84–1.01)	0.074
No		1	

## Discussion

The 2009 pandemic influenza A (H1N1) virus was a novel infectious agent to humans [2]. In this study, we conducted three consecutive serosurveys in 2010 to investigate the temporal trends of seroprevalence to pH1N1 among vaccinated and non-vaccinated populations in Guangdong. We aimed to estimate the effect and implementation of the free pH1N1 vaccination program, to estimate the recurrence risk of the next pandemic wave, and explore influencing factors.

Our results demonstrat that seroprevalence to pH1N1 following the 2009 pandemic among the Guangdong population was 25.1%, including 49.0% of the vaccinated people and 22.8% of the non-vaccinated people. The overall seropositive rate declined to 18.4% in March (survey 2) and rebounded to 21.4% in September (survey 3). Given that seroprevalences among the vaccinated subjects did not differ significantly across the three surveys, the wave for the overall seroprevalance may be due to fluctuations in the natural infection rate with similar activity pattern among non-vaccinated subjects.

Although no difference in seroprevalance was observed during the three surveys periods, demographic analysis of the vaccinated subjects showed that there was a significant growth in seroprevalence in the capital-city group from January to March, which was offset by the reduction in the urban population (people living in urban areas in middle and small-sized cities) from March to September. The growth of seroprevalence in the capital-city may be due to a free pH1N1 vaccination program that had been implemented since October 2009 in Guangdong. Due to a lack of detailed data, we are unable to make a definite inference about the progress of the program in Guangdong. However, it is possible that the main part of the vaccination program in urban areas in the middle and small-sized cities ended before February 2010, whereas the program in the capital-city stopped between March and September 2010 because seroprevalence with HI titer≥ 1∶160 decreased significantly during this period. Therefore, the free vaccination program lasted longer in capital-city areas than in urban areas. In accordance with this, there was a marked drop back in seroprevalence in the urban population. It has been reported in China that the antibody positive rate of pH1N1 in vaccinated people would reach a maximum (81%) at day 30 (calculated since the first day of vaccination) and then began to drop[18]–[20]. It is likely that the decline of the seroprevalence in urban areas was a result of the quick drop in antibody levels among the population from this group. However, we cannot exclude the involvement of other influencing factors in developing this wave of seroprevalence.

Among non-vaccinated subjects, seroprevalence declined significantly from 22.8% to 14.3% between January and March. This is consistent with the data from Guangdong Notifiable Disease Database, which showed that the number of reported pH1N1 cases per day decreased gradually between January and March. A previous study in China also found that antibody positive rates of pH1N1 in patients would reach a maximum (100%) at day 30 (calculated since the first day of onset of the disease) and then begin to drop [18]. So the decline in seroprevalence from January to March is probably due to the quick decrease in protective antibodies in old cases and the reduction of newly reported cases. Such a decline was found among all evaluated demographic groups except for children scattered, teachers, and medical personnel. Teachers and medical personnel are in close contact with high risk population such as students and patients, which would increase their exposure to the H1N1 virus [21].

Seroprevalence in non-vaccinated subjects increased significantly by 26.6% from March to September. This is inconsistent with the report from GD Notifiable Disease Database, in which the number of reported H1N1 cases fluctuated at a low level between March and September. However, it is similar to findings from Outpatient Influenza-like Illness Sentinel Surveillance System which demonstrated a small peak in ILI% (percentage of visits for influenza-like illness) in July 2010, with the proportion for influenza A (H1N1) among ILI positive samples collected from sentinel hospitals also increasing progressively since late June and reaching a small peak (approximately 40%) in late July. The reason for the inconsistency between GD Notifiable Disease Database and Sentinel Surveillance System is that the Notifiable Disease Database is based on passive reporting by health care providers and is less sensitive when compared to active sentinel and serologic surveillance.

Although 26∼60 and >60 years age groups increased their seroprevalences between March and September, the multivariate analysis found that the school-aged population and young adults (6∼15 and 16∼25 years) had a higher risk of infection by pH1N1 for those whose seroprevalences remained highest in the three serosurveys. This is consistent with the studies [21], [22] from the 2009 pandemic period that suggests the notion that school aged children constitute the main conduit for the spread of influenza due to generally higher levels of contact in school. The finding that people in the capital city had higher risk of infection by pH1N1 than other regions also accords with the studies from the 2009 pandemic period, which is explained by more frequent social contact and greater population density in the capital city.

The association between the investigation order of the surveys and the prevalence of pH1N1 indicates that the temporal trends of seroprevalence among the non-vaccinated population are related to other factors as well as age and region. The most probable influencing factor we speculate is the vaccination strategy which was the most effective control measure taken in 2009–2010 by Guangdong authorities because vaccinated people play a barrier role in sustainable human-to-human transmission among the non-vaccinated population. As noted earlier, the free pH1N1 vaccination program began in October 2009 and ended before September 2010 in Guangdong. The free pH1N1 vaccination program helped restrain the possible summer wave of 2010 in Guangdong, but abandoning the program too soon resulted in the slight rebounding of seroprevalence in September 2010.

Several studies reported that previous seasonal influenza vaccinations were associated with higher HI titers against H1N1 [23]–[25]. In our study, seroprevalence in the two groups (with or without seasonal influenza vaccination) were similar. We found a greater decrease in the seropositive rate in the seasonal influenza vaccination group between January and March. Further studies are needed to focus on the relationship between seasonal influenza vaccination history and the seropositive rate to pH1N1.

According to the three surveys, approximately 39.5% (389/985), 44.9% (271/603) and 55.2% (420/731) of the seropositive individuals (non-pH1N1-vaccinated) did not have flu-like symptoms (one or more symptoms) since May 2009. The multivariate analysis did not find a correlation between flu-like symptoms and seroprevalence to pH1N1. Nevertheless, the role of asymptomatic individuals in the spread of 2009 H1N1 cannot be discounted because it has important implications in formulating public health policy that are instituted at ports of entry and at educational institutions during the first pandemic wave and it underscores the need for vigilance at both the community and individual levels to control the spread of disease.

### Limitations

It is important to note several limitations of this study. Firstly, there was no pre-pandemic or early pandemic collection as a baseline which would have been useful to estimate the attack rate or seroprevalence of pH1N1 infection after the 2009 wave of pH1N1 infection. as the seropositivity would decline after 30 days post-infection, the surveys collected in January and September 2010 may have already been too late to capture the pandemic peak. Secondly, the lack of data on indicators of influenza-like illness (such as hospital admission rates), vaccination rates and the period during which vaccinations took place in the region resulted inability to link results to these factors more directly. Finally, though we recorded a high percentage of the seropositive individuals who did not report flu-like symptoms, this may be partly due to potential recall bias.

### Conclusion

In Guangdong, seroprevalance to pH1N1 decreased firstly and then rebounded modestly during the first 9 months following the 2009 pandemic wave of the disease. The free pH1N1 vaccination program carried out smoothly since October 2009 maintained stability with the seroprevalances of the three surveys among the vaccinated population and helped restrain a possible summer wave in 2010 among the non-vaccinated population. Our results suggest that the prevalence of pH1N1 among the non-vaccinated population still correlated with age and population density during the post-pandemic period. An early end of the free pH1N1 vaccination program might be another important reason for the slight rebound in seroprevalance to pH1N1 in 2010. Our study findings can help Guangdong authorities make evidence-based decisions about a long-term vaccination strategy and boost immunity in specific population groups (such as children, capital city residents) to prevent further transmission in the future.
